# Urban vertical farming: innovation for food security and social impact?

**DOI:** 10.1098/rstb.2024.0154

**Published:** 2025-09-18

**Authors:** Alana Kluczkovski, Philip Hadley, Christopher Yap, Ulrike Ehgartner, Bob Doherty, Katherine Denby

**Affiliations:** ^1^ Department of Biology, University of York, York YO10 5DD, UK; ^2^ School for Business and Society, University of York, York YO10 5DD, UK; ^3^ Centre for Food Policy, City St George's University of London, London EC1V 0HB, UK

**Keywords:** controlled environment agriculture, food systems, vertical farming, urban agriculture

## Abstract

Urban vertical farming (VF) has emerged as a potential solution to improve food security and safety for urban populations, as well as to transform wider food systems (FS) to ensure greater sustainability. Existing literature has highlighted both direct and indirect benefits from VF to individuals and communities through novel technology alongside social entrepreneurial innovation. These include the creation of green jobs, and greater access to fresh, healthy food produced locally, as well as community development programmes and avenues for civic participation. We explore relevant literature to critically examine the socio-economic impact of VF, drawing out key issues of debate, while identifying areas of future research and recommendations for practice. We draw attention to critical accounts that have highlighted a need to consider the role of technology within social and political processes. Studies have noted key challenges to VF in achieving social and economic benefits to urban populations, as well as in contributing to food security. Examining VF as an intervention within a wider political economy enables a more rigorous exploration of social impact. A research, policy and practice focus beyond production and business model design is needed to situate VF within broader efforts to transform FS.

This article is part of the theme issue ‘Transforming terrestrial food systems for human and planetary health’.

## 1. Introduction

The scale of the challenges facing our food systems (FS) demands new approaches to growing, processing and distributing healthy and sustainable food for all. Escalating challenges related to population growth, urbanization, climate change and diminishing arable land necessitate urgent and innovative solutions to secure food for future generations [1]. The United Nations (UN) estimates that the global population will exceed 9 billion by 2050 [2], with the majority of this growth concentrated in urban areas, especially in secondary cities and smaller urban centres [3]. In 2022, food insecurity affected 26 and 28% of adults living in urban and peri-urban areas, respectively [4].

Conventional agriculture faces several well documented challenges, including inefficient resource use, substantial greenhouse gas emissions, and significant environmental degradation from practices such as deforestation, over-fertilization and pesticide application [5,6]. These practices also contribute to soil erosion and loss of arable land, limiting long-term productivity [7]. Globally, agriculture is responsible for approximately 70% of all freshwater withdrawals, while also being vulnerable to climate change impacts such as extreme weather and changing rainfall patterns [8]. These environmental drawbacks are coupled with systemic inequities in food distribution and access, exacerbating global food insecurity, particularly in low-income populations and regions most affected by climate change [4]. As global food demand continues to rise—projected to increase by 60% from 2005 to 2050—there have been increasing calls for the adoption of advanced technology in agricultural production [9–12].

Rapid changes in urban populations represent a further significant challenge to the supply and accessibility of fresh food [3]. Urban agriculture (UA) has the potential to contribute towards addressing these challenges. The term is used to refer to a diversity of practices ranging from intensive commercial farming to informal livelihood strategies. UA has been associated with a wide range of positive outcomes at multiple scales, including health and well-being of urban inhabitants [13], providing ecosystem services, and fostering community-building [14,15]. However, a growing body of critical literature has highlighted the potential of UA to contribute to gentrification and displacement, to entrench social inequalities, and to reinforce neoliberal ideas regarding the responsibilities of individuals and the state [16–20].

In recent years, indoor vertical farming (VF) has received attention as a promising approach to UA that has the potential to contribute to the supply of fresh food while fostering a range of social, economic and environmental benefits in urban settings. Indoor VF is a form of controlled environment agriculture (CEA) [21]. By operating in controlled environments, vertical farms can potentially mitigate the risks posed by climate variability, pests and pollution [22] and offer a healthier setting for crop growth [23,24]. VF, which utilizes vertical space through columnar structures or stacked layers, has the potential to improve resource efficiency and reduce the environmental impact of food production. As such, it may offer a more sustainable and resilient approach to urban food security, providing stable, year-round food production [25].

While the environmental benefits and technical dimensions of VF have been a primary focus of research, the ways that VF may contribute to a wider range of interconnected FS challenges, particularly social and economic challenges, are less well understood. Broader literature has highlighted a need to understand technological innovation within wider social and technical transitions [26], as well as a need to place such transitions within an analysis of political economy, politics and inequalities in FS [27,28]. In research, policy and practice, this body of literature points to a need for a critical approach to charting pathways for transforming FS [29,30].

In this article, we critically review existing evidence to examine the potential of VF to contribute towards addressing a range of critical social and economic challenges. Our aim is not to restate the case for VF; rather, it is to situate the rise of VF within broader understandings of the socio-economic impact of UA. In doing so, the article contributes to understanding the role of VF within the wider suite of transformations necessary in our FS as well as the conditions under which VF may contribute towards addressing multiple, interconnected societal challenges.

## 2. Methodology

The methodology for this narrative review draws on recent studies (adapted from [31]) to explore the current and potential socio-economic impact of VF as a form of CEA, as well as to identify areas of future research and recommendations for practice. The studies included were selected through keyword searches conducted on PubMed in October 2023, February 2024 and April 2024. The search terms used, individually and in combinations of up to three terms, included: urban farm, urban agriculture, city farms, urban farming, controlled environment agriculture, precision farming, sustainable agriculture, vertical farming, indoor urban farming, indoor urban agriculture, precision agriculture, social impact, community impact, social well-being impact, and variations thereof. Titles of over 300 papers were initially screened, and 151 relevant scientific publications were selected for this review. The types of articles considered included books and documents, meta-analyses, reviews and systematic reviews.

Studies were included in the reading if they: (i) reviewed the current state of UA, indoor farming or specific aspects of it; (ii) contained unique research and findings not typically found in other reviews, especially with regard to the social impact of urban food production; (iii) addressed emerging topics such as food justice, vertical farms, innovative technologies, and advanced cultivation methods; (iv) discussed combinations and integrations of current technologies and practices; or (v) examined broader aspects of urban food production, including logistics, economics, social and environmental impacts, implementation and policy.

Additionally, further studies were identified by examining the references and citations of the most relevant papers and authors to ensure a thorough understanding of the current state and future potential of urban indoor agriculture. The literature reviewed included peer-reviewed articles, books, conference proceedings, technical documents, technical bulletins, project reports, and commercial websites.

## 3. Situating controlled environment agriculture and vertical farming within the field of urban agriculture

UA can be defined as the cultivation, processing, distribution and marketing of food and other agricultural products within urban areas primarily to feed the local population [3,32]. Typically, UA is not only located within cities but also embedded within urban processes and dynamics [33]: it utilizes urban resources (land, labour, organic wastes, water); it is influenced by urban policies, spatial constraints and market conditions; and it has the potential to impact urban populations and the urban environment.

Over the past 25 years, governmental support for UA has grown at the international, national and local levels, despite some institutional reluctance to incorporate it into broader urban planning [34,35]. Some countries have adopted national policies that promote urban horticulture, including Argentina, Brazil and Cuba [36,37]. The UN has also recognized UA as a key strategy for achieving the Sustainable Development Goals [22,38].

UA encompasses a diverse variety of practices, from neighbourhood allotment gardens to intensive commercial production units, and from rooftop gardens to environment-controlled farms [39]. Each type represents a function of multiple influences, including business models, governance structures, and socio-economic context, and can be associated with a diversity of social and economic impacts.

The current and potential multi-dimensional significance of UA is well documented [40–42]. Some scholars have emphasized the contribution of UA to the health of urban inhabitants [43,44] and to sustainable livelihoods [45], and the therapeutic benefits of urban food growing [46]. Others have emphasized the circular integration of UA into the urban environment through, for example, the re-use of grey water [47], providing ecosystem services [48], and closing the nutrient loop [49].

This latter strand of the UA discourse resonates closely with the more recent discourse on CEA systems, which have been heralded as a form of UA that has the potential to make a significant contribution to the long-term sustainability of cities for its advanced technological approach and potential for significant, high-impact changes [21].

CEA farms are characterized as either enclosed or closed depending on factors such as sun exposure, farming techniques, irrigation methods, size, density, level of control, layout, building type, location and purpose [50]. Enclosed systems include greenhouses; closed systems include indoor farms and vertical farms that use light-emitting diodes (LEDs) instead of sunlight [51–54]. CEA systems protect crops from external weather and regulate microclimates to produce higher, year-round yields [21]. Many of these systems use advanced technology for comprehensive monitoring and automation to maintain optimal environmental conditions and improve energy management [51,55,56].

CEA systems can be established in a wide range of facilities such as warehouses, shipping containers or empty buildings [57,58]. They range from very small mobile systems to large, highly sophisticated systems in high-rise buildings [52]. Both enclosed and closed systems are primarily soil-less and can achieve water savings of up to 95% compared with conventional farming [10,58,59]. While greenhouses are typically located away from urban areas, verticla farms can be situated in urban or peri-urban locations, which can reduce food miles, CO_2_ emissions from transportation, and food waste in the supply chain [60].

VF represents an area of rapid innovation and expansion within CEA [61]. It is an example of an enclosed CEA system. Using advanced irrigation systems such as hydroponics or aeroponics, VF can potentially produce food more sustainably than conventional agriculture by reducing the need for water, fertilizers and pesticides [23] and reducing industrial pollution [25]. VF uses only about 10% of the water required in conventional farming [50].

Vertical farms can be categorized based on their size and purpose. Each category utilizes LED lights, irrigation systems, and vertically stacked shelves or towers for growing various plants. These shelves/towers typically feature computer-controlled growth management systems, allowing users to monitor all systems remotely. Key differentiating factors are their structure, location and mobility, encompassing purpose-built or repurposed urban buildings, modular mobile units, in-store or rooftop systems, and small-scale appliances integrated into homes or offices (table 1).

**Table 1.** Typology of vertical farms

category	characteristics
building-based vertical farms	constructed in either purpose-built or repurposed buildings in urban areas; they include growing plants with artificial light in a shielded space like a factory [1,50,57,58,62].
shipping-container vertical farms	modular and potentially mobile farms built in repurposed shipping containers, which typically measure 2.44 m (w) × 2.59 m (h) and vary in length between 3.05 and 12.19 m [50,58,62]
in-store vertical farms	farms located within the place of consumption or purchase, e.g. retail or restaurants [52,57]; this includes units located within or on the rooftops of both old and new buildings, including commercial and residential structures [63,64].
appliance farms	mobile farm appliances that may be integrated into homes or offices [52,57]

Advocates of VF argue that it can address vulnerability to climate change-induced weather events, including their effect on agricultural land and the global economy [21,65–67]. By reducing the need for conventional farming’s substantial fossil fuel consumption and minimizing food miles (the distance food travels to reach urban consumers), VF can enhance sustainability and efficiency in food production [66]. Moreover, indoor growth systems protect plants from adverse weather and climate change by allowing year-round production and making it feasible to grow crops in harsh environments, where conventional farming is difficult [57].

Proponents have suggested that producing just 10% of the food consumed in urban areas through VF could reduce CO_2_ emissions sufficiently to foster technological advancements that may lead to long-term improvements in biosphere health [25,68]. Despommier [69] further argues that transitioning to VF could alleviate the environmental pressures of conventional agriculture, enabling natural ecosystems to recover and flourish. These studies suggest potential broad impacts, but lack detail on the specific nature of these improvements (e.g. the level of CO_2_ emissions VF could reduce). While assessing the environmental impact of VF is not the aim of this paper, it is important to note that VF has received criticism in existing literature owing to the high energy requirements needed to support CEA systems, including lighting and temperature. These requirements represent potential trade-offs to the potential environmental benefits suggested in VF literature [51,70,71]. However, studies applying life cycle analysis suggest that integrating renewable energy sources, such as solar or wind, into VF systems could significantly reduce these environmental impacts, potentially making VF more competitive with other farming methods [72,73].

While the current and potential contribution of VF and CEA systems to urban sustainability has been increasingly asserted, less attention has been given to analysing how such contributions correspond with the wider benefits and limitations associated with UA. A substantial body of evidence has demonstrated the contribution of more traditional and community-based methods of UA to urban food security, in terms of both contributing to food supply and generating household income [42,45,74,75]. Some studies indicate that UA could play a significant role in feeding cities in the Global North [76] and the Global South [77]. However, other studies have exposed the difficulty, if not impossibility, of accessing the amount of land required for UA to make a significant contribution to a city’s food demands [78].

The past two decades have seen the rise of a discourse that focuses on the social significance and potential of UA. UA has been linked closely with community-building, particularly for marginalized urban groups [79,80]. Urban community gardens, for example, have been celebrated for their capacity to foster diverse communities [81,82] and engage children and young people in community projects [83].

In some contexts, this social impact has extended into the political sphere [84]. Staeheli *et al.* [85], for example, describe the formation of a counter-public in community gardens in New York, through which marginalized urban inhabitants develop alternative visions of urban management. In this vein, other studies have identified the potential for UA to be a socially transformative activity [86].

Yet there is also recognition that the positive social and economic impact of UA can be overstated. The literature on urban farming has highlighted the potential of multiple social benefits, including contributions to food security, public health, skill building and jobs, community development and FS change, leading to an association of UA with food justice [87]. However, there is a need to examine whether socioeconomically disadvantaged communities benefit. It is also evident that UA alone cannot address the fundamental causes of food injustice, which include economic disparities, poverty, and historical and structural racism [19]. Some projects may even perpetuate existing inequities [19].

Urban community gardens can, for example, become exclusionary spaces [88]. Scholars have recognized contradictory politics—a dialectical tension [18]—at the heart of UA [89]. While UA can open new spaces for participation, enhance claims to public space and support access to healthy and affordable food, it can also exacerbate existing dynamics of social, spatial and economic marginalization [90,91], through, for example, ecological gentrification [92]. These authors draw attention to a need to consider systemic conditions, including poverty, low wages and income disparity, that produce food insecurity [93,94].

A significant body of critical literature has explored the limitations of UA for escaping or operating beyond neoliberalism [95,96]. However, Ghose & Pettygrove [97] argue that in the USA, urban community gardens simultaneously contest and reinforce neoliberal practices. Ernwein [98] has argued that UA simultaneously contests the neoliberalization of urban space while reproducing a neoliberal governmentality. The implication across this scholarship is that while UA can deliver significant benefits, it risks entrenching notions of individualism, minimizing the responsibility of the state in FS change and reinforcing structural inequalities [99–101].

## 4. The current and potential socio-economic impacts of vertical farming

This section reviews the evidence for the social and economic impacts of VF. Importantly, across much of the academic discourse, VF practices are rarely disaggregated into the categories identified in table 1. Further analysis that examines the social and economic impacts of different forms of VF represents an important area for further research. We organize this section around three key areas of current and potential impacts that have received most of the scholarly attention: contributions to urban food security through enhanced access to healthy food, contributions to an inclusive urban economy and contributions to urban civic life. Within each section, we reflect on the extent to which evidence regarding the socio-economic impacts of VF aligns with the impacts, opportunities and challenges identified within the wider UA discourse.

### (a) Contributions to urban food security

Existing literature highlights VF as a key innovation for improving urban food security [21,23,64,102,103]. In densely populated areas, VF can maximize production in confined spaces, enhancing the availability of healthy fresh food [102,104,105]. Further benefits of produce from VF relate to food safety and quality, enhanced owing to a controlled indoor environment and minimal use of pesticides and herbicides [3,21,25,64,106]. However, much of this literature identifies pathways to potential impact on urban food insecurity rather than evidencing impacts to date. To some extent, this is likely to reflect the recent proliferation of VF and the more gradual emergence of an academic discourse. It may also reflect a significant gap in terms of the types of research studies that are conducted in relation to vertical farms.

In terms of prevailing international definitions of food and nutrition security [107]—organized in terms of the four pillars of access, availability, utilization and stability—VF has the potential to make significant contributions to the physical availability of food and food stability over time. For example, scholarship has drawn attention to the potential role of VF in supporting the transformation of so-called food deserts, urban neighbourhoods and rural towns without ready access to fresh, healthy and affordable food [108]. In this context, vertical farms and CEA systems more broadly have the potential to ensure greater availability of food and a more stable food supply, through efficient growing cycles, protection from variations in weather and other growing conditions and their ability to operate outside of growing seasons [3]. However, Carolan & Hale [16] have argued that this perceived stability is not necessarily a long-term solution; rather, it only addresses existing gaps in otherwise unjust and unsustainable food value chains.

The contribution of VF to the other pillars of food security—access to food and food utilization—is less straightforward. High initial investment costs [52,70] as well as high running costs associated with VF mean that produce can be limited in range and is often priced relatively highly and less accessible to low-income groups. Currently, the relatively high production costs mean that vertical farms focus on rapidly growing crops such as leafy greens, microgreens and herbs to remain as cost-effective as possible [70,109,110]. Marketing these crops as premium products—emphasizing their traceability, pesticide-free status, freshness and local production—can enhance their economic viability [52,57,111,112] but also raise the price for consumers.

This is particularly important in the context of the intersecting drivers and manifestations of urban inequality [113], such that expanding UA will not automatically improve food security. As low-income communities are likely already subject to ‘underinvestment and discriminatory patterns’, vertical farms are vulnerable to falling into a corporate FS model of ‘profit maximization and resource use efficiency’, in which social justice-oriented practices are sidelined [114]. A number of studies have also shown a concentration of urban farms in places where they are not most needed to address food insecurity, linked with the role of urban farming in greening areas, which Yuan *et al.* [100] argue risks making them a tool in gentrification ‘to make neighbourhoods more attractive to the upper class’. An increase in property prices through such urban development is one example given as needing consideration in exploring the social aspects of urban farming.

High production costs can potentially be mitigated in certain climates and through technological advancements [54]. For example, vertical farms are reported to be more efficient in regions where heating requires more electricity than lighting, such as at higher latitudes, or in areas where water is scarce and energy is more affordable, such as parts of the Middle East, where water-use-efficient vertical farms may be more desirable [54,111]. However, this review has not found evidence of vertical farms that produce healthier and more sustainable food than conventional agriculture at a lower price to direct consumers than existing, supermarket-dominated value chains.

Cost of food, especially healthy fresh produce, is often in tension with other high costs of living in urban areas, causing low-income residents to become dependent on emergency food services and food pantries [114]. Seigner *et al.* [114] highlight affordability challenges to urban-produced food and cite examples of urban farms donating to food banks, but caution the lack of scholarship on the consumption or impact of these donations.

As indicated above, it has been argued that vertical farms could play a key role in addressing the food security of poor and ethnically diverse urban neighbourhoods that lack access to affordable, healthy food options [115,116]. This may be more likely where vertical farms are small-scale and built, for example, in unused buildings that have been repurposed [25]. However, there is a lack of empirical cases documenting this in practice.

This is not to say that VF cannot contribute to urban food security. Rather it means that the pathways of impact are distinct from other forms of urban food production, for example, scholarship from the Global South has emphasized the contribution of UA to food security through the development of additional household income [100], while scholarship in allotments and other forms of urban community gardens from the Global North have emphasized contributions to food security through subsistence, social education and communal utilization of healthy foods [100]. This diversity of pathways to food security through UA points to the need for a diversification of approaches to meet the diversity of needs of different urban inhabitants, of which VF is likely to be one important approach.

### (b) Contributions to an inclusive urban economy

VF literature has highlighted the role of innovative technology together with a viable business modelin creating both direct and indirect impacts, such as ground-breaking new food supply and distribution networks, while also addressing food security and environmental challenges [117]. Some of these potential economic and social impacts include the creation of new, much-needed green collar jobs, from farm nursery management and resource procurement to IT, office personnel and other areas [25]. Further jobs, literature suggests, will be created in grocery stores, organic food markets and local distribution and transportation networks [21,116]. However, as above, these impacts are highlighted as potential contributions, rather than being arrived at through an analysis of the impact of VF. Such assertions of impact, including job creation, are also made largely without consideration of inequities within FS and the wider political economies in which vertical farms exist [118].

The conceptual focus placed upon an interlinked triumvirate of novel technology, viable new business models and agricultural productivity also concurs with mainstream approaches to FS in research, policy and practice. What unites dominant visions is adherence to a predominantly productionist perspective, which focuses on the need to significantly increase food production and calorie availability, through production efficiencies, capital investments and new technologies [118]. In contrast to the *a priori* privileging of production and novel technology, critical studies have drawn attention to the need to understand VF (and FS interventions) as both technical and social transitions [26,119].

To understand and identify the pathways through which VF may contribute to inclusive urban economies, it is crucial to broaden the discussion to consider, for example, Who benefits from VF projects, and how? Such questions are, however, left relatively unexplored in the mainstay of existing and emerging literature. Instead, studies on VF have emphasized its potential to create not only jobs, but new business ventures, while leveraging automated technology to aid the development of smart cities by providing locally sourced food, reducing the need for extensive transportation networks, and promoting efficient use of space [63,64,67,117,120,121]. Further contributions noted include the attraction of VF to a younger generation, owing to its intensive use of advanced technologies in controlling indoor environments [122,123]. Moreover, it is argued that the involvement of this generation may drive further innovation in agricultural technology.

Rather than providing a socio-economic analysis, much literature tends to follow a tautological approach, in which the wider impact of VF is attested to by its innovative nature and presumed advantages in agricultural production and environmental impact. For example, Biancone *et al.* [117] argue that VF may be a solution to urban food security through spreading new technologies and improved engagement with local economies. In ensuring that vertical farms are such a solution, emerging literature has highlighted the key role of novel and viable business models along with entrepreneurship [124–127]. The focus of analysis among some studies has therefore explored the nature of successful business models in VF. One such study has concluded that entrepreneurship, for instance, should aim for an efficient growing system aided with technologies in achieving environmental and economic sustainability goals [117]. Organizational design, as these authors argue, is critical to building innovative business models to ensure VF can deliver a socially sustainable supply of food and the development of transformative technologies [128].

Literature on VF has thus emphasized a need for new approaches, for innovations, techniques and processes for both food production and consumption [129]. As such an innovation, VF is presented by this body of literature as constantly improving and expanding and as changing the way food is produced and consumed [129]. One example of this is modular, small-scale vertical farms that make significant use of automation and can be set up in a range of settings, such as restaurants, residential areas and supermarkets [129]. The value of these ‘growing-service systems’, argues one review, rests on intangibles such as fresher products, local production and automated control. In this manner, VF firms are experimenting by adapting business models and enabling sustainable development as a goal [129].

Emerging VF literature has argued that, as both a technological and entrepreneurial innovation, VF has the potential to disrupt and decentralize the conventional FS by operating across different scales and locations, while increasing local decision-making autonomy [130,131]. Some authors have associated UA more broadly with contributing to a redistribution of resources and power by commoning urban resources, for example, by turning urban wastelands or interstitial areas into community-managed farmland [119,131,132]. However, in our review, we find that these studies do not provide a pathway for, or a sufficiently critical analysis of how, such redistribution might be achieved through VF, and neither do they provide sufficient empirical evidence. In addition, studies that have highlighted the potential game-changing role of VF have also noted that many VF firms across North America, Europe and the Middle East have yet to fully address broader sustainability goals beyond environmental impacts, with little focus on social or economic sustainability [129]. There is also a dearth of literature on specific case studies of vertical farms of different scales [129]. Despommier [64], a key proponent of VF, has reflected that it is unclear whether VF would be successful globally from an economic and/or social perspective, since the concept is still too new.

While much of the existing and emerging literature on VF makes implicit or, in some cases, explicit references to direct individual or indirect community/collective social and economic benefits, a smaller body of studies has taken a more critical approach [133,134]. One review exploring the potential of scaling up VF has noted that the contextual conditions required for VF to be sustainable have not yet been holistically assessed [119]. These authors used a multi-level perspective approach to analyse VF as part of a socio-technical transition, examining three levels: *niches*, *landscapes* and *regimes* [26,119]. 'Niches' refers to potential novelties and social innovations that may challenge the dominant regime, including VF, but also alternative food networks and community agriculture. 'Landscapes' refers to broader processes that exert pressure on the prevailing regime, potentially enabling transformation. 'Regimes' represent the existing structures comprising various actors, market forces, technologies, policies and cultural and industrial norms [26,119]. For these authors, the dominant regime is the current FS, while the landscape of broader processes includes the approaching food crisis and globalized neoliberal capitalism [119]. Specific examples of landscape pressures include climate change, which exacerbates agricultural vulnerabilities, and shifting demographic patterns, such as urbanization and population growth, which alter food demand and land use [119]. These broader processes also include industrialized farming, which contributes to biodiversity loss and environmental degradation, extensive food miles leading to higher carbon emissions, increasing corporate involvement in UA, and the growth-oriented focus of neoliberal political economy [117].

Scaling up VF, argue Petrovics & Giezen [119], requires the optimal combination of contextual factors. This emphasizes the importance of understanding the particular elaboration of niches, landscapes and regimes, such as the role of VF investment schemes like venture capitalism in North America, and their implications for land ownership and ownership of the means of food production and intellectual property in VF operations. Carolan [135] has argued that capital-intensive large-scale VF in North America provides a short-term ‘fix’, potentially shortens food supply chains, but gives unsustainable and unjust systems a new lease of life until the next crisis. These authors argue for a need to consider the systemic issues that necessitate interventions such as VF and caution that, without a reflection on VF as a social and technical intervention, VF could lead to further commodification of agricultural products, further segregation between the experience of food and its modes of production, and its catering to the wealthy [16,87,119,135].

From an inclusive urban economy perspective, some studies have raised important concerns about the actual impact of UA on skill building, education, and community development [87]. These authors point out that urban farms often struggle to provide sufficient living-wage jobs, rely heavily on unpaid labour, and face financial instability due to dependency on grants, donations and off-farm income [19,87,136–138]. Moreover, the context and specifics of UA projects influence who benefits, with advantages often accruing to property owners and new residents more than disadvantaged groups, while UA may also be situated within processes of gentrification [87]. To identify a pathway for how VF as a form of UA may contribute to inclusive urban economies, it is therefore critical to situate it within the broader political economy of urban development. This requires moving beyond the focus of much existing literature—the technicalities of agricultural production and the presumed role of technological and entrepreneurial innovation [87,100]. There is a need for both rigorous analysis of socio-economic impact and deeper attention to questions of power in FS interventions and transformations [87,139–141].

### (c) Contributions to urban civic life

Alongside urban food security and economic impacts, literature has noted the role of VF in community development, with studies pointing to a wide range of potential benefits, including outreach programmes, education, establishing business centres, as well as linking with social workers and facilitating community advocacy in urban governance [116,142]. Through the involvement of social workers, one study has argued that interdisciplinary teams can address issues of food access and sustainable production in low-income neighbourhoods [116]. Social participation through education, training and community engagement programmes, alongside the sale of healthy food locally, argues another study, can have economic, social and health benefits [142]. Through such social entrepreneurial and technological innovation, vertical farms, some literature has suggested, can even become sites for community advocacy and civic virtue development, as citizens are empowered to engage with urban planners, public authorities and decision-makers [116].

While such contributions to urban civic life through VF may be possible, as above, our review has not found studies that present empirical evidence of such contributions. Instead, literature has focused on an implicit or explicit presentation of the role of technological and entrepreneurial innovation. To leverage multiple environmental, economic and social benefits, the prevailing approach in the VF literature has highlighted the way new business models are being developed, for example to provide functions and services instead of traditional products [129]. Social and economic impacts, including the role of such innovation regarding existing dynamics in FS and wider political economies, are largely presumed rather than analysed. Wider literature on UA has, however, drawn attention to the inevitable intersection between urban farming and social injustices as projects develop [100,135]. In one study, authors note the need for deliberate processes of community inclusion in urban decision-making to address social injustices, through which mutual relations can be built between public officials and civil society. Rather than a singular focus on urban farming, such processes include local government structures, as well as food policy councils, local activists and community organizations [100,135].

As a note of caution, some authors have argued that not all UA practitioners connect food cultivation to political values or actions, and in those conditions, UA is unlikely to function as a mechanism for food democracy, other movements for social justice, or structural change [87,136]. On a related point, one review has noted that not all UA planning efforts seek to help disadvantaged residents suffering from food injustice [87]. Furthermore, although urban farming projects such as community gardens have been praised for fostering mental health, civic participation and community pride, the benefits are not always equitably distributed [87]. Broadening out a focus on urban civic life to the role of UA in addressing food injustice experienced by disadvantaged communities, Horst *et al.* [87] argue that UA by itself is unable to address the structural causes of inequities in FS and is more fairly viewed as one possible strategy among an array of others, including poverty alleviation, in seeking greater food justice.

Horst *et al.* [87] concluded that UA should not be viewed as a panacea but instead as one potential intervention among an array of strategies that may enhance food justice but only if the benefits accrue to urban residents who experience food injustice and insecurity. Similarly, based on their research, Petrovics & Giezen [119] highlighted the limitations of single socio-technical interventions and argued that owing to the complexity of urban reality, VF should not be assessed from the perspective of single supply-end interventions. In considering how to scale up VF, one challenge these authors note is a common focus on the role of technology as independent of social and political processes [87,119].

To understand the contribution of VF to urban civic life, it is therefore necessary to understand VF not only as a transformation of technological processes, but as a phenomenon with transformative power in the societal sphere [87,119]. To understand such transformation, contextual spatial factors, power relationships and the productive nature of political struggles are important to consider [87,119]. If VF becomes a key aspect of urban development, it is even more important not only to question who benefits and how benefits can be justly distributed, but also to situate these questions within a critical analysis of the relationship between power and innovation in FS [28,135].

A structured framework to synthesize the advantages and disadvantages from the three potential impact categories identified is presented in table 2. This emphasizes the tension between potential and realized impacts in each category, highlighting the need for more empirical evidence, nuanced approaches and policies addressing equity and inclusion.

**Table 2.** Advantages and disadvantages of urban vertical farming in the three potential impact categories emerging from the literature. CEA, controlled environment agriculture; FS, food systems; UA, urban agriculture; VF, vertical farming.

impact category	advantages	disadvantages
urban food security	— potential to increase food availability and stability of food supply through CEA (efficient growing systems and year-round production) — potential to transform food deserts by increasing the availability of healthy, fresh food — controlled indoor environments improve food quality and safety, reducing the need for pesticides — protection from climate and seasonal variability, potentially enhancing food stability	— limited evidence for impact on urban food insecurity, including access to food and food utilization — high production costs make produce less accessible to low-income populations — risk of aligning with corporate models focused on profit rather than food justice — limited crop range (e.g. leafy greens) owing to economic constraints — potential role in gentrification, raising property prices and excluding marginalized groups — perceived increases in food supply stability and availability mask unsustainable and unjust food value chains
inclusive urban economy	— potential creation of green jobs across agriculture, distribution, IT and retail, alongside new food supply networks — potential for entrepreneurial and technological innovation to transform conventional FS: opportunities for small-scale farms and new business models — potential to attract younger generations through technological innovation — possibility for VF to disrupt conventional FS and foster resource sharing	— limited empirical evidence of job creation or impact on food supply networks, alongside evidence of low wages and unpaid labour in urban farming — presumed role and impact of technological and entrepreneurial innovation versus a lack of socio-economic analysis and evidence — lack of research on VFs of different scales and sizes — economic benefits may favour property owners or wealthier individuals over marginalized communities — risks of prioritizing capital-intensive VF projects that replicate unsustainable systems — high dependency on grants or donations in small-scale urban farming projects — may perpetuate segregation between food consumers and producers, focusing benefits on affluent markets — few case studies evaluate long-term social and economic sustainability goals
urban civic life	— potential for community-building through education, training and outreach programmes — potential opportunities for community advocacy in governance and urban planning — potential for economic, social and health benefits through integrating community engagement programmes with the sale of healthy food locally — potential for promoting civic participation through citizen engagement	— limited evidence of impactful contributions to community advocacy or civic life — risk of perpetuating social inequities if community participation is not explicitly integrated into projects — focus of VF literature being on technical and entrepreneurial innovation cf. socio-political analysis of urban inequities — lack of deliberate inclusion processes risks excluding marginalized voices in urban planning — inability of VF or UA projects by themselves to address structural causes of inequities in FS

## 5. Conclusions: opportunities to enhance the contribution of vertical farming to food systems transitions

Much existing and emerging literature on VF has focused on analysing the production aspects of this novel and evolving technology [59,124]. Alongside this literature, studies have sought to explore VF as an innovative business model [25,117]. In both cases, the focus of research is on defining and delineating effective technological and organizational features for successful VF operations. In reference to both novel technology and organizational design, VF as an entrepreneurial innovation is credited with a series of agricultural production, economic and environmental benefits, alongside a number of social benefits. As discussed, a smaller body of literature has sought to question this panacea presentation of VF as a solution not only to agricultural challenges, but to urban development, food security and societal challenges more widely [87,116,119,135]. It is also the case that much existing literature has focused on North America, as well as on large-scale VF operations.

We hope that this review is timely, as technical innovations in VF and CEA are increasingly matched by the strength of desire across societies to trial new approaches to food production, distribution and consumption, as well as scholarly and policy interest. Our contribution is to highlight the limits of any one approach or set of technologies in isolation and to advocate for a critical and evidence-led approach to enabling equitable socio-technical transitions within our FS.

Our review has highlighted a number of *potential* social benefits associated with VF, both to individuals and to wider urban communities. These include the creation of green jobs and local food supply chains, and greater access to healthy fresh food (transforming food deserts), leading to health and economic benefits. However, crops produced in vertical farms are generally sold as premium products. In addition, literature has pointed to community development work and potential avenues for civic participation, as well as integration of these activities with public and community services [116]. Most of these social benefits are listed as potential (unevidenced) benefits within existing literature, and rest on a conceptual presumption concerning the role of social entrepreneurial innovation in agricultural production and business model design in urban areas, as well as the presumed direct and indirect impacts on urban populations. Critical studies have highlighted challenges to these benefits, including corporate investment models, gentrification, unaffordability or inaccessibility of VF produce to low-income communities, as well as low wage levels among urban agricultural workers and a reliance on unpaid labour. Finally, community development and civic participation activities can be both inclusionary and/or exclusionary.

Our review has highlighted the need to consider the role of pre-established socio-economic structures [100], policy contexts and the wider FS when seeking to understand the social impact of VF, especially beyond the individual level to wider community benefits. This requires examining VF as a socio-technical intervention [26], as well as considering the broader political economy and particular urban context in which vertical farms are implemented. Much VF literature to date reflects a technocratic, productionist perspective typical of mainstream FS analysis, where power dynamics and institutional frameworks are taken as given, rather than socially constructed [143]. VF may form part of wider efforts to transform FS, but as Anderson & Leach reflect [28], rather than technical transitions, the need is for ‘deeper transformations’ for global FS and for sustainability and equity more broadly. Such transformation is inevitably profoundly political, requiring power and political economy ‘to be addressed head-on’ [11,27,28,117,144,145].

There is therefore a need for further research exploring the social implications of VF of different models, scales and geographical locations, especially small-scale and outside North America. Such research may also critique the artificial separation of VF technology from its social, economic and political context. In this sense, the key question may not necessarily be to explore the social impact of VF but to understand the way in which VF develops through organizational, social, economic and political relations. In contrast to much of the research that has examined the socio-economic impacts of VF, future research could productively focus on the interactions between VF and its context, unpacking the specific local conditions that enable the positive potential impacts of VF to be realized. Avenues of research may explore VF organizational development beyond a focus on business model design [117] to the ebb and flow of VF innovation, including efforts to create social impact or to engage communities, from a political ecology or critical institutional perspective [146]. Such a focus on the interactions between VF practices and their contexts is vital for better understanding the contribution of VF to more equitable and sustainable FS within a broad range of emerging technological, organizational and policy innovations. Beyond these broad research areas, specific knowledge gaps also remain regarding, for example, attitudes (including issues of desirability and cultural appropriateness) towards the types of foods predominantly produced by VF by low-income groups.

Suggested research approaches to explore the social implications and impact of VF include community-based participatory and ethnographic methodologies, longitudinal case studies and political economy analysis of FS interventions (drawing on existing work [28]). Building on studies that have sought to qualify the prevailing panacea-based approach to VF in literature, policy and practice, adopting a critical focus on VF development and impact may help to explore why, despite an emphasis on effective organizational structures [25], social benefits and impact is often difficult to ascertain and define (or achieve). Exploring the way in which VF is embedded within wider political economies as a socioecological innovation should provide a key step in this direction.

## Data Availability

This article has no additional data.

## References

[1] Tilman D, Clark M. 2014 Global diets link environmental sustainability and human health. Nature **515**, 518–522. (10.1038/nature13959) 25383533

[2] United Nations, Department of Economic and Social Affairs, Population Division. 2024 World population prospects 2024. See https://www.unpopulation.org/.

[3] Orsini F, Kahane R, Nono-Womdim R, Gianquinto G. 2013 Urban agriculture in the developing world: a review. Agron. Sustain. Dev. **33**, 695–720. (10.1007/s13593-013-0143-z)

[4] FAO, IFAD, UNICEF, WFP, WHO. 2023 The state of food security and nutrition in the world 2023. Rome, Italy: Food and Agriculture Organization. (10.4060/cc3017en)

[5] Foley JA *et al*. 2011 Solutions for a cultivated planet. Nature **478**, 337–342. (10.1038/nature10452) 21993620

[6] Intergovernmental Panel on Climate Change (IPCC). 2022 Technical summary. In Climate change and land (eds PR Shukla, J Skea, R Slade, AA Khourdajie, R van Diemen, D McCollum, M Pathak, S Some), pp. 37–74. Cambridge, UK: Cambridge University Press.

[7] Montgomery DR. 2007 Soil erosion and agricultural sustainability. Proc. Natl Acad. Sci. USA **104**, 13268–13272. (10.1073/pnas.0611508104) 17686990 PMC1948917

[8] David M. 2013 Water for food, water for life: a comprehensive assessment of water management in agriculture. London, UK: Taylor and Francis.

[9] De Oliveira FJB, Ferson S, Dyer R. 2021 A collaborative decision support system framework for vertical farming business developments. Int. J. Decis. Support Syst. Technol. **13**, 1–33. (10.4018/ijdsst.2021010103)

[10] Barbosa G, Gadelha F, Kublik N, Proctor A, Reichelm L, Weissinger E, Wohlleb G, Halden R. 2015 Comparison of land, water, and energy requirements of lettuce grown using hydroponic vs. conventional agricultural methods. Int. J. Environ. Res. Public Health **12**, 6879–6891. (10.3390/ijerph120606879) 26086708 PMC4483736

[11] Leach M, Nisbett N, Cabral L, Harris J, Hossain N, Thompson J. 2020 Food politics and development. World Dev. **134**, 105024. (10.1016/j.worlddev.2020.105024)

[12] Alexandratos N, Bruinsma J. 2012 World Agriculture towards 2030/2050: the 2012 revision. See https://www.fao.org/agrifood-economics/en/.

[13] Bellows AC, Brown K, Smit J. 2004 Health benefits of urban agriculture. Portland, OR: Community Food Security Coalition’s North American Initiative on Urban Agriculture. See https://community-wealth.org/content/health-benefits-urban-agriculture.

[14] Middle I, Dzidic P, Buckley A, Bennett D, Tye M, Jones R. 2014 Integrating community gardens into public parks: an innovative approach for providing ecosystem services in urban areas. Urban For. Urban Green. **13**, 638–645. (10.1016/j.ufug.2014.09.001)

[15] Camps-Calvet M, Langemeyer J, Calvet-Mir L, Gómez-Baggethun E. 2016 Ecosystem services provided by urban gardens in Barcelona, Spain: insights for policy and planning. Environ. Sci. Policy **62**, 14–23. (10.1016/j.envsci.2016.01.007)

[16] Carolan M, Hale J. 2016 'Growing' communities with urban agriculture: generating value above and below ground. Community Dev. **47**, 530–545. (10.1080/15575330.2016.1158198)

[17] DeLind LB. 2015 Where have all the houses (among other things) gone? Some critical reflections on urban agriculture. Renew. Agric. Food Syst. **30**, 3–7. (10.1017/s1742170513000525)

[18] McClintock N. 2014 Radical, reformist, and garden-variety neoliberal: coming to terms with urban agriculture’s contradictions. Local Environ. **19**, 147–171. (10.1080/13549839.2012.752797)

[19] Pdxscholar P, McClintock N, Mahmoudi D. 2016 Socio-spatial differentiation in the sustainable city: socio-spatial differentiation in the sustainable city: a mixed-methods assessment of residential a mixed-methods assessment of residential gardens in metropolitan Portland. Oregon, USA Gardens in Metropolitan Portland, Oregon, USA. See https://pdxscholar.library.pdx.edu/usp_fac.

[20] Reynolds K. 2015 Disparity despite diversity: social in justice in New York City’s urban agriculture system. Antipode **47**, 240–259. (10.1111/anti.12098)

[21] Despommier D. 2019 Vertical farms, building a viable indoor farming model for cities. Field Actions Sci. Rep. **2019**, 68–73. http://journals.openedition.org/factsreports/5737

[22] United Nations. 2015 Transforming our world: the 2030 Agenda for Sustainable Development. New York, NY: United Nations. See https://sdgs.un.org/publications/transforming-our-world-2030-agenda-sustainable-development-17981.

[23] Healy RG, Rosenberg JS. 2011 Land use and the states, 2nd edn. New York, NY: RFF Press. (10.4324/9781315064406)

[24] Mukherji N, Morales A. 2010 Zoning for urban agriculture. Zoning Practice **3-10**, 2–7. https://www.planning.org/publications/document/9006942/

[25] Al-Kodmany KM, Al-Kodmany K. 2016 Sustainable tall buildings cases from the Global South. Int. J. Architect. Res. ArchNet-IJAR **2**, 52–66. (10.26687/archnet-ijar.v10i2.1054)

[26] Geels FW. 2011 The multi-level perspective on sustainability transitions: responses to seven criticisms. Environ. Innov. Soc. Transit. **1**, 24–40. (10.1016/j.eist.2011.02.002)

[27] Oliver TH *et al*. 2018 Overcoming undesirable resilience in the global food system. Glob. Sustain. **1**, e9. (10.1017/sus.2018.9)

[28] Anderson M, Leach M. 2019 Transforming food systems: the potential of engaged political economy. IDS Bull. **50** 131–146. (10.19088/1968-2019.123)

[29] Dentoni D, Waddell S, Waddock S. 2017 Pathways of transformation in global food and agricultural systems: implications from a large systems change theory perspective. Curr. Opin. Environ. Sustain. **29**, 8–13. (10.1016/j.cosust.2017.10.003)

[30] Dentoni D, Bitzer V, Schouten G. 2018 Harnessing wicked problems in multi-stakeholder partnerships. J. Bus. Ethics **150**, 333–356. (10.1007/s10551-018-3858-6)

[31] Weidner T, Yang A, Hamm MW. 2019 Consolidating the current knowledge on urban agriculture in productive urban food systems: learnings, gaps and outlook. J. Clean. Prod. **209**, 1637–1655. (10.1016/j.jclepro.2018.11.004)

[32] Fletcher EI, Collins CM. 2020 Urban agriculture: declining opportunity and increasing demand—how observations from London, UK, can inform effective response, strategy and policy on a wide scale. Urban For. Urban Green. **55**, 126823. (10.1016/j.ufug.2020.126823)

[33] Mougeot L. 2000 Urban agriculture: definition, presence, potentials, and risks. In Growing cities, growing food: urban agriculture in the policy agenda (eds N Bakker, M Dubelling, S Gundel), pp. 1–42. Feldafing, Germany: DSE.

[34] Cissé O, Fatou N, Gueye D. 2005 Institutional and legal aspects of urban agriculture in French-speaking West Africa: from marginalization to legitimization. See http://www.centredakar.org/.

[35] Food and Agriculture Organization of the United Nations (FAO). 2001 Urban and peri-urban agriculture: a briefing guide for the successful implementation of urban and peri-urban agriculture in developing countries and countries of transition, SPFS/DOC/27.8 Rev.2, 1st edn. Rome, Italy: FAO, Special Programme for Food Security. See https://www.fao.org/fileadmin/templates/FCIT/PDF/briefing_guide.pdf.

[36] van Veenhuizen R (ed). 2006 Cities farming for the future: urban agriculture for green and productive cities. Leusden, The Netherlands: RUAF Foundation, International Institute of Rural Reconstruction (IIRR), and International Development Research Centre (IDRC). See https://ruaf.org/document/cities-farming-for-the-future-urban-agriculture-for-green-and-productive-cities/.

[37] Doernberg A, Horn P, Zasada I, Piorr A. 2019 Urban food policies in German city regions: an overview of key players and policy instruments. Food Policy **89**, 101782. (10.1016/j.foodpol.2019.101782)

[38] Semenova R, Wilhelm K. 2021 Sustainable development goals addressed by urban farming. Interreg North-West Europe: GROOF European Regional Development Fund, European Union. See http://www.groof.eu/.

[39] URBES Project. Urban agriculture: landscapes connecting people, food and biodiversity (urbes factsheet no. 7). Stockholm, Sweden: URBES – Urban Biodiversity and Ecosystem Services. See https://oppla.eu/sites/default/files/old_files/uploads/urbesfactsheet07web2.pdf (accessed 30 July 2025).

[40] Mougeot L. 2005 Agropolis: the social, political and environmental dimensions of urban agriculture. London, UK: Earthscan.

[41] Poulsen MN, McNab PR, Clayton ML, Neff RA. 2015 A systematic review of urban agriculture and food security impacts in low-income countries. Food Policy **55**, 131–146. (10.1016/j.foodpol.2015.07.002)

[42] Redwood M. 2008 Agriculture in urban planning: generating livelihoods and food security. London, UK: Earthscan.

[43] Brown KH, Jameton AL. 2000 Public health implications or urban agriculture. J. Public Health Policy **21**, 20–39. (10.2307/3343472) 10754796

[44] Hodgson K, Caton Campbell M, Bailkey M. 2011 Urban agriculture: growing healthy, sustainable places. Washington, DC: American Planning Association.

[45] Hoornweg D, Munro-Faure P. 2008 Urban agriculture for sustainable poverty alleviation and food security. Rome, Italy: Food and Agriculture Organization of the United Nations. See https://www.fao.org/fileadmin/templates/FCIT/PDF/UPA_-WBpaper-Final_October_2008.pdf.

[46] O’Brien D. 2010 Cultivating our garden. In Gardening – philosophy for everyone: cultivating wisdom(eds F Allhoff, D O’Brien), pp. 192–203. Hoboken, NJ: Wiley‑Blackwell. (10.1002/9781444324563.ch14)

[47] Pinderhughes R. 2004 Alternative urban futures. Lanham, MD: Rowman and Littlefield Publishers.

[48] Lin BB, Philpott SM, Jha S. 2015 The future of urban agriculture and biodiversity-ecosystem services: challenges and next steps. Basic Appl. Ecol. **16**, 189–201. (10.1016/j.baae.2015.01.005)

[49] Mougeot LJA. 2006 Growing better cities: urban agriculture for sustainable development. Ottawa, Canada: International Development Research Centre.

[50] Chatterjee A, Debnath S, Pal H. 2020 Implication of urban agriculture and vertical farming for future sustainability. In Urban horticulture - necessity of the future. London, UK: IntechOpen. (10.5772/intechopen.91133)

[51] Vatistas C, Avgoustaki DD, Bartzanas T. 2022 A systematic literature review on controlled-environment agriculture: how vertical farms and greenhouses can influence the sustainability and footprint of urban microclimate with local food production. Atmosphere **13**, 1258. (10.3390/atmos13081258)

[52] Butturini M, Marcelis LFM. 2020 Vertical farming in Europe. In Plant factory: an indoor vertical farming system for efficient quality food production, 2nd edn (eds T Kozai, G Niu, M Takagaki), pp. 77–91. Amsterdam, The Netherlands: Elsevier. (10.1016/B978-0-12-816691-8.00004-2)

[53] Esmaeli H, Roshandel R. 2020 Optimal design for solar greenhouses based on climate conditions. Renew. Energy **145**, 1255–1265. (10.1016/j.renene.2019.06.090)

[54] Graamans L, Baeza E, van den Dobbelsteen A, Tsafaras I, Stanghellini C. 2018 Plant factories versus greenhouses: comparison of resource use efficiency. Agric. Syst. **160**, 31–43. (10.1016/j.agsy.2017.11.003)

[55] Engler N, Krarti M. 2021 Review of energy efficiency in controlled environment agriculture. Renew. Sustain. Energy Rev. **141**, 110786. (10.1016/j.rser.2021.110786)

[56] Benis K, Reinhart CF, Ferrão P. 2017 Building-integrated agriculture (BIA) in urban contexts: testing a simulation-based decision support workflow. In Proc. 15th Int. Conf. Int. Building Performance Simulation Assoc. (Building Simulation 2017), San Francisco, 7–9 August 2017 (eds CS Barnaby, M Wetter), pp. 2337–2346. San Francisco, CA: International Building Performance Simulation Association. (10.26868/25222708.2017.479)

[57] Van Gerrewey T, Boon N, Geelen D. 2021 Vertical farming: the only way is up? Agronomy **12**, 2. (10.3390/agronomy12010002)

[58] Chole AS, Jadhav AR, Shinde VN. 2021 Vertical farming: controlled environment agriculture. Just Agric. **1**, 055. https://www.justagriculture.in/files/newsletter/2021/jan/055.pdf

[59] Despommier D. 2011 The vertical farm: controlled environment agriculture carried out in tall buildings would create greater food safety and security for large urban populations. J. Verbrauch. Leb. **6**, 233–236. (10.1007/s00003-010-0654-3)

[60] Ramankutty N, Evan AT, Monfreda C, Foley JA. Farming the planet: 1. Geographic distribution of global agricultural lands in the year 2000. Glob. Biogeochem. Cycles **22**, GB1003. (10.1029/2007GB002952)

[61] Birkby J. 2016 Vertical Farming. ATTRA Sustainable Agriculture - A program of the National Center for Appropriate Technology (NCAT). See http://www.attra.ncat.org/.

[62] Chaudhry AR, Mishra VP. 2019 A comparative analysis of vertical agriculture systems in residential apartments. In Advances in Science and Engineering Technology International Conferences (ASET), Dubai, United Arab Emirates, 2019, pp. 1–5. New York, NY: IEEE. (10.1109/ICASET.2019.8714358)

[63] Touliatos D, Dodd IC, McAinsh M. 2016 Vertical farming increases lettuce yield per unit area compared to conventional horizontal hydroponics. Food Energy Secur. **5**, 184–191. (10.1002/fes3.83) 27635244 PMC5001193

[64] Despommier D. Vertical farms in horticulture. See http://www.fao.org/docrep/008/y5800e/Y5800E06.htm.

[65] United Nations, Department of Economic and Social Affairs, Population Division. 2017 World population prospects: the 2017 revision, key findings and advance tables. Working paper no. ESA/P/WP/248. New York, NY: United Nations, Department of Economic and Social Affairs, Population Division. See https://desapublications.un.org/publications/world-population-prospects-2017-revision.

[66] Kalantari F, Mohd Tahir O, Mahmoudi Lahijani A, Kalantari S. 2017 A review of vertical farming technology: a guide for implementation of building integrated agriculture in cities. Adv. Eng. Forum **24**, 76–91. (10.4028/www.scientific.net/aef.24.76)

[67] Muller A, Ferré M, Engel S, Gattinger A, Holzkämper A, Huber R, Müller M, Six J. 2017 Can soil-less crop production be a sustainable option for soil conservation and future agriculture? Land Use Policy **69**, 102–105. (10.1016/j.landusepol.2017.09.014)

[68] Wood S, Sebastian KL, Scherr SJ. 2000 Pilot analysis of global ecosystems: agroecosystems. Washington, DC: World Resources Institute. See https://www.wri.org/research/pilot-analysis-global-ecosystems-agroecosystems.

[69] Despommier D. 2010 The vertical farm: feeding the world in the 21st century, 1st edn. New York, NY: Thomas Dunne Books.

[70] Benke K, Tomkins B. 2017 Future food-production systems: vertical farming and controlled-environment agriculture. Sustainability **13**, 13–26. (10.1080/15487733.2017.1394054)

[71] Arcasi A, Mauro AW, Napoli G, Tariello F, Vanoli GP. 2024 Energy and cost analysis for a crop production in a vertical farm. Appl. Therm. Eng. **239**, 122129. (10.1016/j.applthermaleng.2023.122129)

[72] Schmidt Rivera X, Rodgers B, Odanye T, Jalil-Vega F, Farmer J. 2023 The role of aeroponic container farms in sustainable food systems – the environmental credentials. Sci. Total Environ. **860**, 160420. (10.1016/j.scitotenv.2022.160420) 36435240

[73] Gargaro M, Hastings A, Murphy RJ, Harris ZM. 2024 A cradle-to-customer life cycle assessment case study of UK vertical farming. J. Clean. Prod. **470**, 143324. (10.1016/j.jclepro.2024.143324)

[74] Edmondson JL, Childs DZ, Dobson MC, Gaston KJ, Warren PH, Leake JR. 2020 Feeding a city – Leicester as a case study of the importance of allotments for horticultural production in the UK. Sci. Total Environ. **705**, 135930. (10.1016/j.scitotenv.2019.135930) 31837547

[75] Walsh LE, Mead BR, Hardman CA, Evans D, Liu L, Falagán N, Kourmpetli S, Davies J. 2022 Potential of urban green spaces for supporting horticultural production: a national scale analysis. Environ. Res. Lett. **17**, 014052. (10.1088/1748-9326/ac4730)

[76] Alaimo K, Packnett E, Miles RA, Kruger DJ. 2008 Fruit and vegetable intake among urban community gardeners. J. Nutr. Educ. Behav. **40**, 94–101. (10.1016/j.jneb.2006.12.003) 18314085

[77] Zezza A, Tasciotti L. 2010 Urban agriculture, poverty, and food security: empirical evidence from a sample of developing countries. Food Policy **35**, 265–273. (10.1016/j.foodpol.2010.04.007)

[78] MacRae R, Gallant E, Patel S, Michalak M, Bunch M, Schaffner S. 2010 Could Toronto provide 10% of its fresh vegetable requirements from within its own boundaries? J. Agric. Food Syst. Community Dev. **1**, 105–127. (10.5304/jafscd.2010.012.008)

[79] Cabannes Y, Raposo I. 2013 Peri-urban agriculture, social inclusion of migrant population and right to the city. City **17**, 235–250. (10.1080/13604813.2013.765652)

[80] Smit J, Bailkey M, Van Veenhuizen R. 2006 Urban agriculture and the building of communities. In Cities farming for the future (ed. R Veenhuizen), pp. 145–171. Ottawa, Canada: International Development Research Centre.

[81] Holland L. 2004 Diversity and connections in community gardens: a contribution to local sustainability. Local Environ. **9**, 285–305. (10.1080/1354983042000219388)

[82] Yap C. 2019 Self-organisation in urban community gardens: autogestion, motivations, and the role of communication. Sustainability **11**, 2659. (10.3390/su11092659)

[83] Hung Y. 2004 East New York farms: youth participation in community development and urban agriculture. Child. Youth Environ. **14**, 56–85. (10.1353/cye.2004.0027)

[84] Yap C, Anderson CR. 2025 Learning the city through urban agriculture. Environ. Plann. D, 02637758241304667. (10.1177/02637758241304667)

[85] Staeheli LA, Mitchell D, Gibson K. 2002 Conflicting rights to the city in New York’s community gardens. GeoJournal **58**, 197–205. (10.1023/b:gejo.0000010839.59734.01)

[86] Certomà C, Tornaghi C. 2015 Political gardening. Transforming cities and political agency. Local Environ. **20**, 1123–1131. (10.1080/13549839.2015.1053724)

[87] Horst M, McClintock N, Hoey L. 2017 The intersection of planning, urban agriculture, and food justice: a review of the literature. J. Am. Plann. Assoc. **83**, 277–295. (10.1080/01944363.2017.1322914)

[88] Donald B, Blay-Palmer A. 2006 The urban creative-food economy: producing food for the urban elite or social inclusion opportunity? Environ. Plan. A **38**, 1901–1920. (10.1068/a37262)

[89] Harris E. 2009 Neoliberal subjectivities or a politics of the possible? Reading for difference in alternative food networks. Area **41**, 55–63. (10.1111/j.1475-4762.2008.00848.x)

[90] Meenar MR. 2017 Assessing the spatial connection between urban agriculture and equity. Built Environ. **43**, 364–375. (10.2148/benv.43.3.364)

[91] Wolch JR, Byrne J, Newell JP. 2014 Urban green space, public health, and environmental justice: the challenge of making cities ‘just green enough’. Landsc. Urban Plann. **125**, 234–244. (10.1016/j.landurbplan.2014.01.017)

[92] Dooling S. 2009 Ecological gentrification: a research agenda exploring justice in the city. Int. J. Urban Region. Res. **33**, 621–639. (10.1111/j.1468-2427.2009.00860.x)

[93] Pudup MB. 2008 It takes a garden: cultivating citizen-subjects in organized garden projects. Geoforum **39**, 1228–1240. (10.1016/j.geoforum.2007.06.012)

[94] Weissman E. 2015 Entrepreneurial endeavors: (re)producing neoliberalization through urban agriculture youth programming in Brooklyn, New York. Environ. Educ. Res. **21**, 351–364. (10.1080/13504622.2014.993931)

[95] Guthman J. 2008 Neoliberalism and the making of food politics in California. Geoforum **39**, 1171–1183. (10.1016/j.geoforum.2006.09.002)

[96] Holt-Giménez E. 2010 Food security, food justice, or food sovereignty? Food First Backgr. **16**, 4. https://www.archive.foodfirst.org/publication/food-security-food-justice-or-food-sovereignty/

[97] Ghose R, Pettygrove M. 2014 Urban community gardens as spaces of citizenship. Antipode **46**, 1092–1112. (10.1111/anti.12077)

[98] Ernwein M. 2017 Urban agriculture and the neoliberalisation of what? ACME **16**, 249–275. (10.14288/acme.v16i2.1387)

[99] Walthall B. 2018 Robert Biel 2016: Sustainable food systems: the role of the city. London: UCL Press. Int. J. Urban Reg. Res. **42**, 175–176. (10.1111/1468-2427.12597)

[100] Yuan GN, Marquez GPB, Deng H, Iu A, Fabella M, Salonga RB, Ashardiono F, Cartagena JA. A review on urban agriculture: technology, socio-economy, and policy. Heliyon **8**, e11583. (10.1016/j.heliyon.2022.e11583) PMC9668687 36406682

[101] Yuan GN, Marquez GPB, Deng H, Iu A, Fabella M, Salonga RB, Ashardiono F, Cartagena JA. 2022 A review on urban agriculture: technology, socio-economy, and policy [Erratum]. Heliyon **9**, e13165. (10.1016/j.heliyon.2023.e13165) PMC9668687 36406682

[102] Thomaier S, Specht K, Henckel D, Dierich A, Siebert R, Freisinger UB, Sawicka M. 2015 Farming in and on urban buildings: present practice and specific novelties of zero-acreage farming (ZFarming). Renew. Agric. Food Syst. **30**, 43–54. (10.1017/s1742170514000143)

[103] Corvalán C, Simon H, McMichael A. 2006 Ecosystems and human well-being: health synthesis: a report of the millennium ecosystem assessment. Geneva, Switzerland: WHO.

[104] Bohn K, Viljoen A. The edible city: envisioning the Continuous Productive Urban Landscape (CPUL). See https://www.field-journal.org/.

[105] Magazine UA. 2011 RUAF 10 years. See https://ruaf.org/.

[106] Cho R. 2011 Vertical farms: from vision to reality. See https://news.climate.columbia.edu/2011/10/13/vertical-farms-from-vision-to-reality/.

[107] F.A.O. 2014 Global initiative on food loss and waste reduction. See https://www.fao.org/3/i2776e/i2776e00.pdf.

[108] Cummins S, Macintyre S. 2002 "Food deserts"—evidence and assumption in health policy making. BMJ **325**, 436. (10.1136/bmj.325.7361.436) 12193363 PMC1123946

[109] Dsouza A, Price GW, Dixon M, Graham T. 2021 A conceptual framework for incorporation of composting in closed-loop urban controlled environment agriculture. Sustainability **13**, 2471. (10.3390/su13052471)

[110] Beacham AM, Vickers LH, Monaghan JM. 2019 Vertical farming: a summary of approaches to growing skywards. J. Hortic. Sci. Biotechnol. **94**, 277–283. (10.1080/14620316.2019.1574214)

[111] Allegaert SD. 2019 The vertical farm industry: exploratory research of a wicked situation. Master’s thesis, Wageningen University, The Netherlands. https://library.wur.nl/WebQuery/theses/directlink/2282094.

[112] Benis K, Ferrão P. 2018 Commercial farming within the urban built environment – taking stock of an evolving field in northern countries. Glob. Food Secur. **17**, 30–37. (10.1016/j.gfs.2018.03.005)

[113] Yap C, Cociña C, Levy C. 2021 *The urban dimensions of inequality and equality*. Barcelona, Spain: United Cities and Local Governments (UCLG). See https://openaccess.city.ac.uk/id/eprint/27107/.

[114] Siegner A, Sowerwine J, Acey C. 2018 Does urban agriculture improve food security? Examining the nexus of food access and distribution of urban produced foods in the United States: a systematic review. Sustainability **10**, 2988. (10.3390/su10092988)

[115] Larsen K, Gilliland J. 2008 Mapping the evolution of “food deserts” in a Canadian city: supermarket accessibility in London, Ontario, 1961–2005. Int. J. Health Geogr. **7**, 16. (10.1186/1476-072x-7-16) 18423005 PMC2387138

[116] Besthorn FH. 2013 Vertical farming: social work and sustainable urban agriculture in an age of global food crises. Aust. Social Work **66**, 187–203. (10.1080/0312407x.2012.716448)

[117] Biancone PP, Brescia V, Lanzalonga F, Alam GM. 2022 Using bibliometric analysis to map innovative business models for vertical farm entrepreneurs. Br. Food J. **124**, 2239–2261. (10.1108/bfj-08-2021-0904)

[118] Harris J, Anderson M, Clément C, Nisbett N. 2019 The political economy of food. IDS Bull. **50**, 2. (10.19088/1968-2019.112)

[119] Petrovics D, Giezen M. 2022 Planning for sustainable urban food systems: an analysis of the up-scaling potential of vertical farming. J. Environ. Plann. Manag. **65**, 785–808. (10.1080/09640568.2021.1903404)

[120] King A, Shackleton CM. 2020 Maintenance of public and private urban green infrastructure provides significant employment in Eastern Cape towns, South Africa. Urban For. Urban Green. **54**, 126740. (10.1016/j.ufug.2020.126740)

[121] Katz B, Bradley J. 2013 The metropolitan revolution: how cities and metros are fixing our broken politics and fragile economy Washington, DC: Brookings Institution Press.

[122] Jones JB. 2014 Complete guide for growing plants hydroponically. Boca Raton, FL: CRC Press.

[123] Isaacs A, Parris-Aaron M, Blair R. Hydroponics home based vegetable production system manual. See http://www.iica.int.

[124] Avgoustaki DD, Xydis G. 2020 Indoor vertical farming in the urban nexus context: business growth and resource savings. Sustainability **12**, 1965. (10.3390/su12051965)

[125] Tooy D, Supriatna E, Ma’ruf MI, Parandy LM, Barki K. 2023 Towards global food security: vertical farming as an innovative solution. Endless **6**, 335–347. (10.54783/endlessjournal.v6i3.229)

[126] McClements DJ, Barrangou R, Hill C. 2020 . Annu. Rev. Food Sci. Technol. **12**, 1–30. (10.1146/annurev-food-062220-090105) 33348992

[127] Trimi S, Berbegal-Mirabent J. 2012 Business model innovation in entrepreneurship. Int. Entrep. Manag. J. **8**, 449–465. (10.1007/s11365-012-0234-3)

[128] Al-Chalabi M. 2015 Vertical farming: skyscraper sustainability? Sustain. Cities Soc. **18**, 74–77. (10.1016/j.scs.2015.06.003)

[129] Martin M, Bustamante MJ. 2021 Growing-service systems: new business models for modular urban-vertical farming. Front. Sustain. Food Syst. **5**, 787281. (10.3389/fsufs.2021.787281)

[130] Glaros A, Newell R, Benyam A, Pizzirani S, Newman L. 2024 Vertical agriculture’s potential implications for food system resilience: outcomes of focus groups in the Fraser Valley, British Columbia. Ecol. Soc. **29**. (10.5751/es-14547-290112)

[131] Mancebo F. 2016 Urban agriculture, commons and urban policies: scaling up local innovation. Challenges Sustain. **4**, 10–19. (10.12924/cis2016.04010010)

[132] Pfeiffer A, Silva E, Colquhoun J. 2015 Innovation in urban agricultural practices: responding to diverse production environments. Renew. Agric. Food Syst. **30**, 79–91. (10.1017/s1742170513000537)

[133] Tregear A. 2011 Progressing knowledge in alternative and local food networks: critical reflections and a research agenda. J. Rural Stud. **27**, 419–430. (10.1016/j.jrurstud.2011.06.003)

[134] Forssell S, Lankoski L. 2015 The sustainability promise of alternative food networks: an examination through ‘alternative’ characteristics. Agric. Human Values **32**, 63–75. (10.1007/s10460-014-9516-4)

[135] Carolan M. 2022 It’s about time: temporal and spatial fixes find vertical farms and local food in the shadow of COVID-19. J. Peasant Stud. **49**, 1446–1465. (10.1080/03066150.2022.2082287)

[136] Reynolds K, Cohen N. 2016 Beyond the kale: urban agriculture and social justice activism in New York City. Athens, GA: University of Georgia Press.

[137] Biewener C. 2016 Paid work, unpaid work, and economic viability in alternative food initiatives: reflections from three Boston urban agriculture endeavors. J. Agric. Food Syst. Community Dev. **6**, 35–53. (10.5304/jafscd.2016.062.019)

[138] Dimitri C, Oberholtzer L, Pressman A. 2016 Urban agriculture: connecting producers with consumers. Br. Food J. **118**, 603–617. (10.1108/bfj-06-2015-0200)

[139] Alkon AH, Cadji J. 2020 Sowing seeds of displacement: gentrification and food justice in Oakland, CA. Int. J. Urban Region. Res. **44**, 108–123. (10.1111/1468-2427.12684)

[140] Walker S. 2016 Urban agriculture and the sustainability fix in Vancouver and Detroit. Urban Geogr. **37**, 163–182. (10.1080/02723638.2015.1056606)

[141] Safransky S. 2014 Greening the urban frontier: race, property, and resettlement in Detroit. Geoforum **56**, 237–248. (10.1016/j.geoforum.2014.06.003)

[142] Prasetiyo WH, Budimansyah D, Roslidah N. 2016 Urban farming as a civic virtue development in the environmental field. Int. J. Environ. Sci. Educ. **11**, 3139–3146.

[143] de Schutter L. 2019 Bioeconomy transitions through the lens of coupled social-ecological systems: a framework for place-based responsibility in the global resource system. Sustainability **11**, 5705. (10.3390/su11205705)

[144] Scoones I, Leach M, Newell P (eds). 2015 The politics of green transformations. In The politics of green transformations pp. 1–24. London, UK: Routledge. (10.4324/9781315747378-1)

[145] Scoones I *et al*. 2020 Transformations to sustainability: combining structural, systemic and enabling approaches. Curr. Opin. Environ. Sustain. **42**, 65–75. (10.1016/j.cosust.2019.12.004)

[146] Cleaver F. 2012 Development through bricolage: rethinking institutions for natural resource management. London, UK: Routledge. (10.4324/9781315094915)

